# Morpho-radiological and brain endocast analysis in the study of *Hyperostosis Frontalis Interna* (HFI): A combined approach

**DOI:** 10.1371/journal.pone.0281727

**Published:** 2023-03-06

**Authors:** Elena Varotto, Francesco Pio Cafarelli, Francesca Maglietta, Cícero Moraes, Pietrantonio Ricci, Francesco Maria Galassi

**Affiliations:** 1 Archaelogy, College of Humanities, Arts and Social Sciences, Flinders University, Adelaide, SA, Australia; 2 FAPAB Research Center, Avola (SR), Sicily, Italy; 3 Department of Clinical and Experimental Medicine, University of Foggia, Foggia, Apulia, Italy; 4 Ortogonline Treinamento em Desenvolvimento Profissional e Consultoria LTDA, Sinop, MT, Brazil; 5 Department of Medical and Surgical Sciences, Institute of Legal Medicine, “Magna Graecia” University, Catanzaro, Italy; 6 Department of Anthropology, Faculty of Biology and Environmental Protection, University of Lodz, Lodz, Poland; University of Florence, ITALY

## Abstract

The purpose of this study is to anatomically evaluate the impact on the patient *intra vitam* of an endocranial condition on a late 20^th^ century skull housed in the Section of Legal Medicine of the University of Foggia (Foggia, Apulia, Italy). After performing a retrospective diagnosis, the condition is framed in the broader context of studies on this pathology. An anthropological and radiological analysis (X-ray and CT scan imaging) made it possible to confirm the preliminary information and to detail the osteological diagnosis of HFI. In order to assess the impact on the cerebral surface of the endocranial growth a 3D endocast was obtained using the Software *OrtogOnBlender*. The skull is demonstrated to have belonged to a female senile individual known, from limited documentary evidence, to have suffered from a psychiatric condition during her life. The final diagnosis is *hyperostosis frontalis interna* (HFI), Type D. Although a direct correlation between the demonstrated intracranial bony growth and the onset of the patient’s psychiatric condition is difficult to retrospectively ascertain, the pressure exerted on this female individual’s frontal lobe may have contributed to further degenerative behavioural changes in the last years of her life. This case adds to previous knowledge, especially from the palaeopathological literature, on this condition and, for the first time, presents a neuroanatomical approach to assess the global impact of the disease.

## Introduction

**Fig 1:**
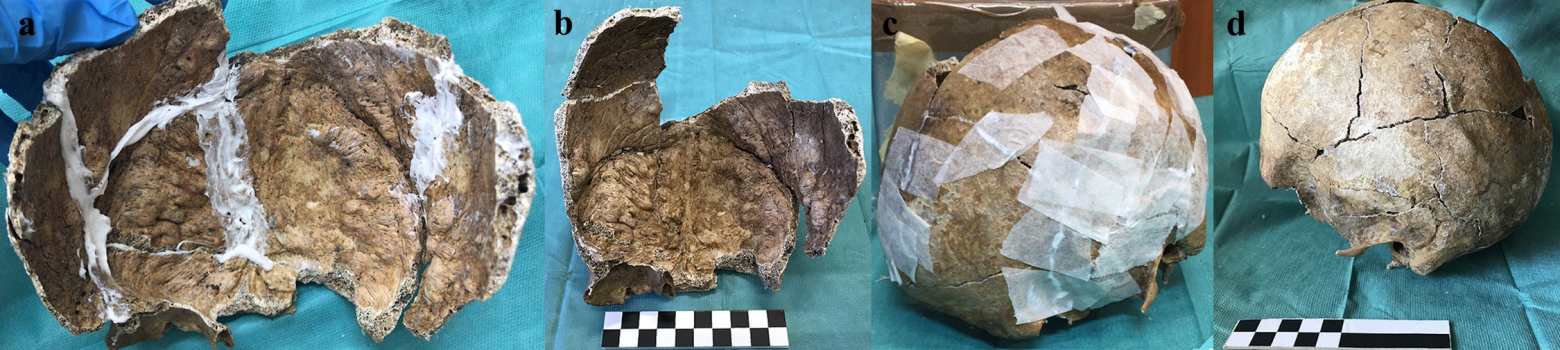
a. The skull at the moment of its discovery; b. The skull during the correct restoration; c. The final stage of the restoration process; d. The completely restored skull.

The Section of Legal Medicine of the University of Foggia (Foggia, Apulia, Italy) has hosted for decades in its archives the fragments of a skull which had been poorly restored with excessive layers of an unspecified type of glue (**Fig 1A**) and in a state of preservation which, given the grayish-brown colour of the cranial surfaces and the presence of some pebbles obstructing some natural cranial foramina, suggested that it may have been buried underground for some time.

This skull has been preserved in the university hospital for research purposes, after being examined during a legal case. Now it belongs to the hospital and is being made available for scientific purposes since no living relatives are known to exist. In the box in which the skull was found there were some notes on the case to which the skull pertained dating back to the 1990, probably left by the medical examiner who first analysed it; on January 1999 this coroner carried out the exhumation of the woman to whom the skull had belonged. The woman, who died about 80 years old and about 7 years before her exhumation took place, had allegedly been affected by a psychiatric illness, although no documental evidence certifying a more precise diagnosis has been found so far.

From the morphological perspective, having noted a peculiar morphology of the endocranial surface of the frontal bone, a multidisciplinary research group analysed this specimen through a full anthropological and radiological study in order to clarify the nature of that visually detected aberrant anatomy.

## Materials and methods

The fragmented skull was washed in order to remove the glue and then newly restored with water-based glue (polyvinyl acetate) with the aim of preserving the bone tissue better and properly reconstruct as much of the skull as possible (**Fig 1B–1D**).

Standard methods used in bioarchaeological and forensic anthropological studies were utilised in order to assess the biological profile from the skull. The sex of the individual to whom the skull belonged was determined using the Ferembach et al. method [1], while age at death was estimated from ectocranial sutures using the Meindl and Lovejoy method [2]. A general visual pathological assessment of the entire skull was performed and compared to similar morphologies described in the relevant literature.

The restored skull was then subjected to a radiological investigation and a 3D virtual reconstruction:

X-ray equipment: OmniDiagnost Eleva (Philips Medical System);
X ray imaging parameters: 80 kV, 20 mAs;
CT-Scan equipment: GE LightSpeed 16 rows;
CT-Scan parameters: helical acquisition with 0.625 mm of thickness, 120 kV, 300 mAs, FOV 24 cm.

The CT scans were used to generate a multiplanar reconstruction (MPR) of the skull and a video (vd. **S1 Video**) using the *OsiriX Lite* version 11.0.3 (Pixmeo Software).

Subsequently, the software *OrtogOnBlender* [3] was used in order to reconstruct the skull from the CT scans and extract the endocast representing the brain sheathed by the encephalic meninges.

*OrtogOnBlender* is an add-on for surgical planning that expands Blender’s capabilities so that it performs tasks that were not originally developed, such as reading, viewing, editing, reconstructing and exporting CT scans. Both of the aforementioned tools are free and open source.

Finally, as typically performed in the multidisciplinary study in human remains [4–7], after a morphological description of the frontal endocranial lesion and the formulation of a retrospective diagnosis, a contextualisation of this case in the broader field of biological anthropological studies was provided.

**Fig 2:**
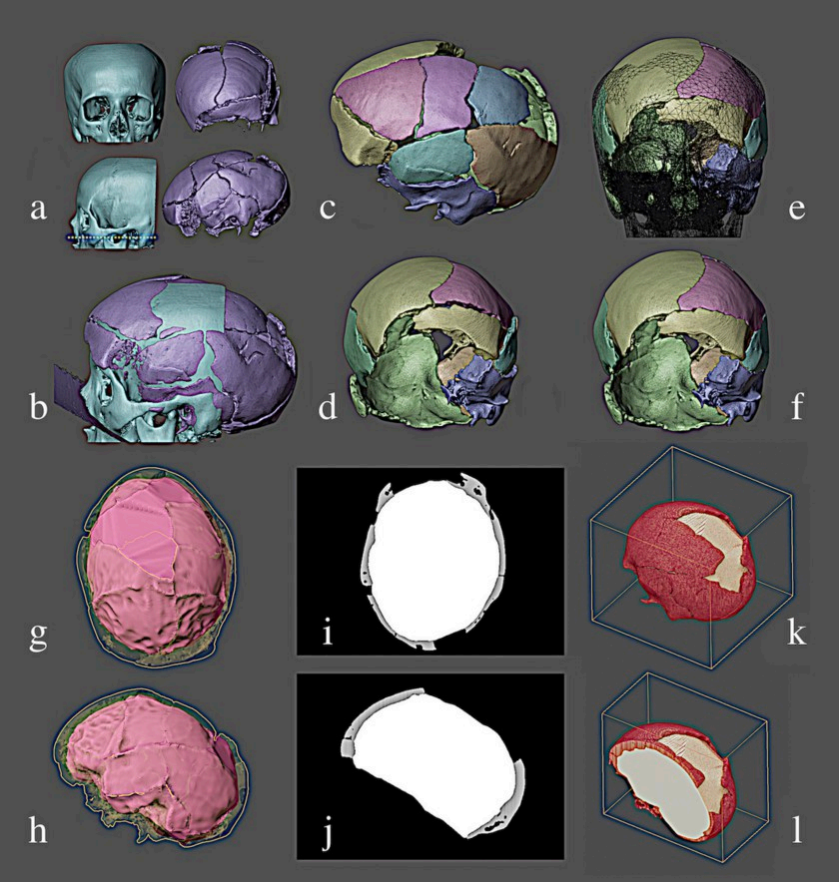
a-l. Steps of the 3D generation of the endocranium.

The tomography was reconstructed in a 3D mesh [8] using the threshold with the value of -300 (**Fig 2A, right side**). A female mature adult skull of European ancestry from a virtual donor (**Fig 2A, left side**) was imported to serve as a reference for the tighter fitting of the pieces, as the skull was broken and slightly deformed by analogical collage. The base skull was segmented into pieces corresponding to the original cracks (**Fig 2C and 2D**) and the pieces were coupled in the best possible fit (**Fig 2F**) respecting the crack structure and the reference of the virtual donor (**Fig 2E**). Once the studied skull was adapted onto the virtual model, the pieces were joined in a Boolean mesh [9]. Through a 3D mesh editing process, the inner shell of the reconstructed skull was used to generate the endocranium [10] (**Fig 2G and 2H**). Once the skull and endocranium model were closed (without holes), an aggregated tomography was created resulting in a DICOM file with different densities for the skull and endocranium volumes (**Fig 2I–2L**).

**Fig 3:**
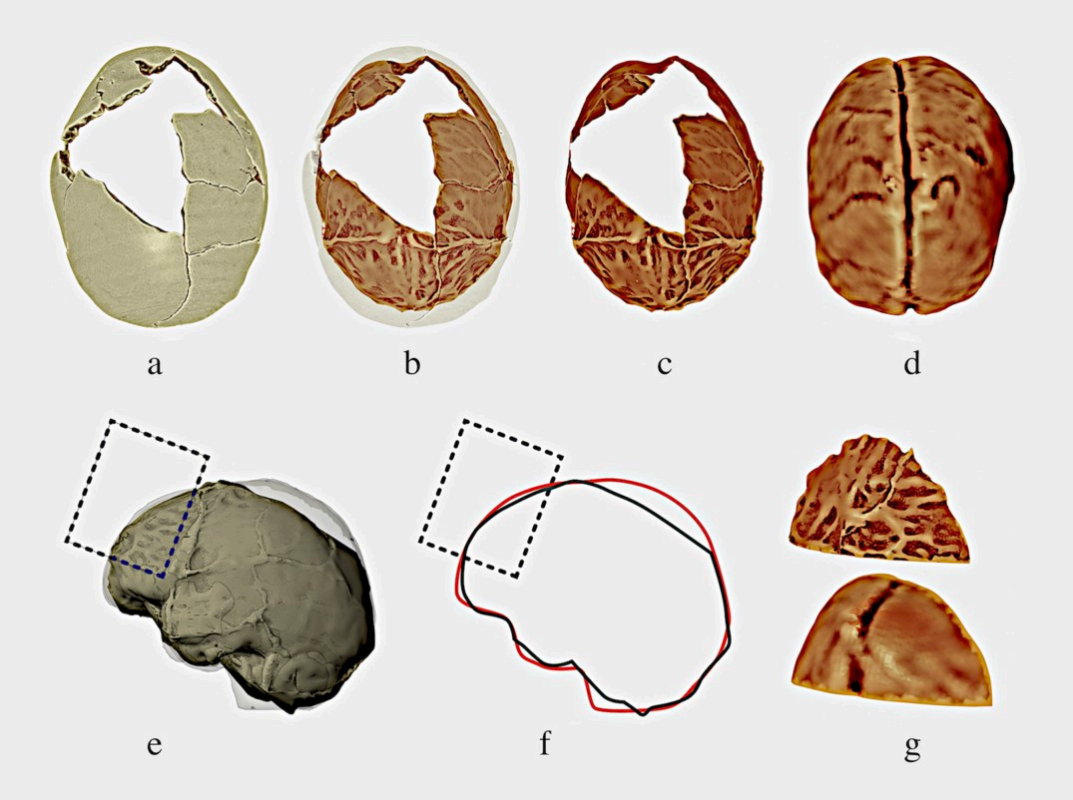
a-g. Brain endocast reconstruction and volume of the brain including the meninges. The area of the frontal lobe affected by the pathological process is indicated by a rectangle.

The skull, as shown above, has some regions missing (**Fig 3A**) and this reflects the generation of an incomplete endocranium (**Fig 3A and 3B**). By way of comparison, an endocranium was created from a complete skull (**Fig 3D**), it was aligned with the incomplete endocranium and as several regions lacked structure, the authors chose part of the frontal surface where in both the structure was complete (**Fig 3E and 3F**). Alignment was performed manually in a digital scene, in order to make the most objective adjustments without any mesh overlap, since it made it possible to make objects transparent, create clipping borders (cuts as in floor plans) and other commands that helped to observe the interlocking of the pieces (links to more information on this technique: https://docs.blender.org/manual/en/latest/render/shader_nodes/shader/transparent.html; https://docs.blender.org/manual/en/2.79/editors/3dview/navigate/clip.html).

Once the region with Boolean calculations was isolated, two objects were generated (**Fig 3G**) whose volumes were calculated.

**Fig 4:**
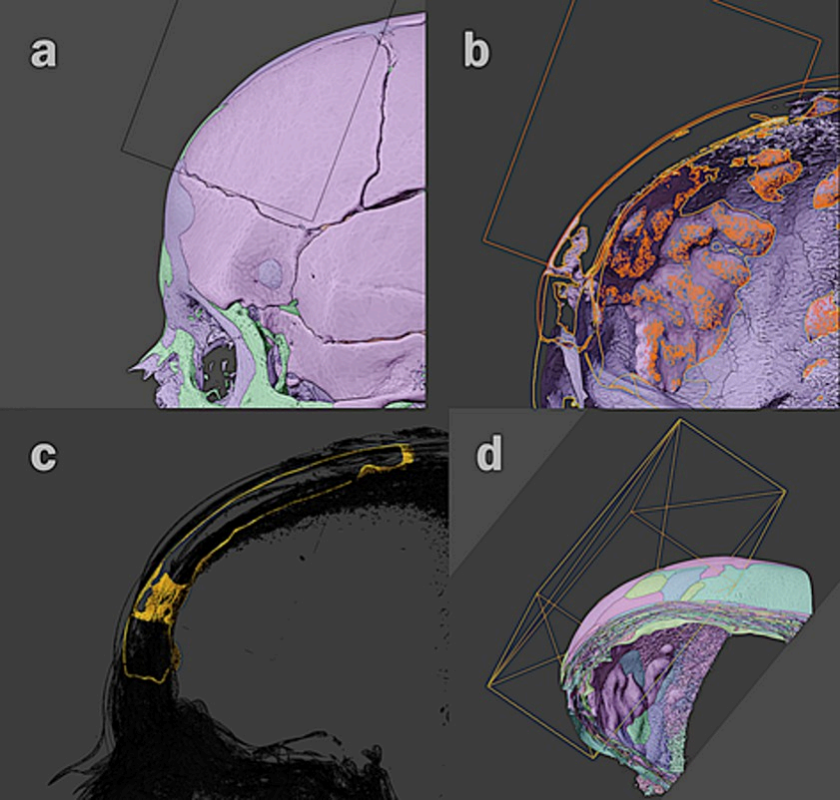
a-d. Phases of the alignment process between the digitised donors’ skulls with the one with the anatomical alteration studied in this article.

In order to have more meaningful results, a larger sample was used for morphological comparison: 18 skulls available for scientific research and anonymised in author C. M.’s tomographic collection were selected, 10 skulls of individuals with European ancestry (Brazilian Europeans) and 8 skulls of Europeans, all belonging to female adults. The skulls, already digitised (**Fig 4A**), were aligned with that with the endocranial alterations (**Fig 4C**), and received the same volumetric segmentation with Boolean calculations applied to the skull with the investigated alteration (**Fig 4B and 4D**).

## Results

Considering the morphological features of the skull, it was determined to have belonged to a female individual, whereas her age category was estimated to be that of a senile (score for sites 1–7 ‘vault’ sutural ages: 21), based on the total closure of the cranial sutures that could be observed.

**Fig 5:**
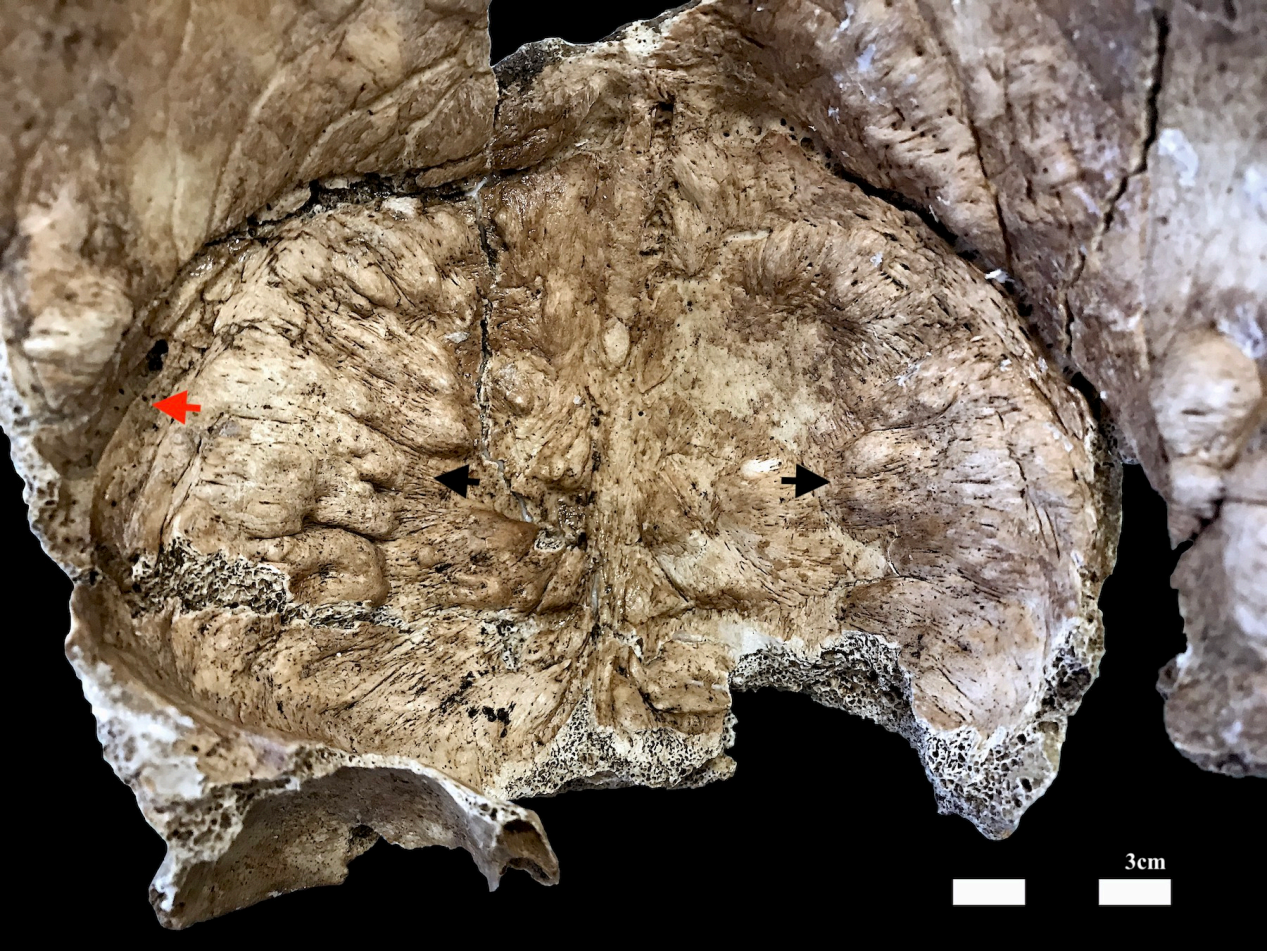
Detail of the bony overgrowth on the endocranial surface of the frontal and parietal bones (black arrows). On the left half of the frontal bone, very close to the coronal suture, it is possible to see the deep groove of the ascending branch of the middle meningeal artery (red arrow).

The ectocranial surface of the skull showed no alterations. However, on the endocranial surface of the skull, an aberrant morphology was detected: the frontal bone, except for the midline (insertion line of the *falx cerebri*), is affected by an irregurlarly elevated surface (more than 50% of the whole area) with clearly demarcated borders having the shape of nodular continuous bony overgrowths. Some smaller nodules can also be found on the parietal surfaces near the coronal suture (**Fig 5**) sparing the grooves for the ascending branches of the middle meningeal arteries: the left one, nonetheless, is deeper than normally found.

**Fig 6:**
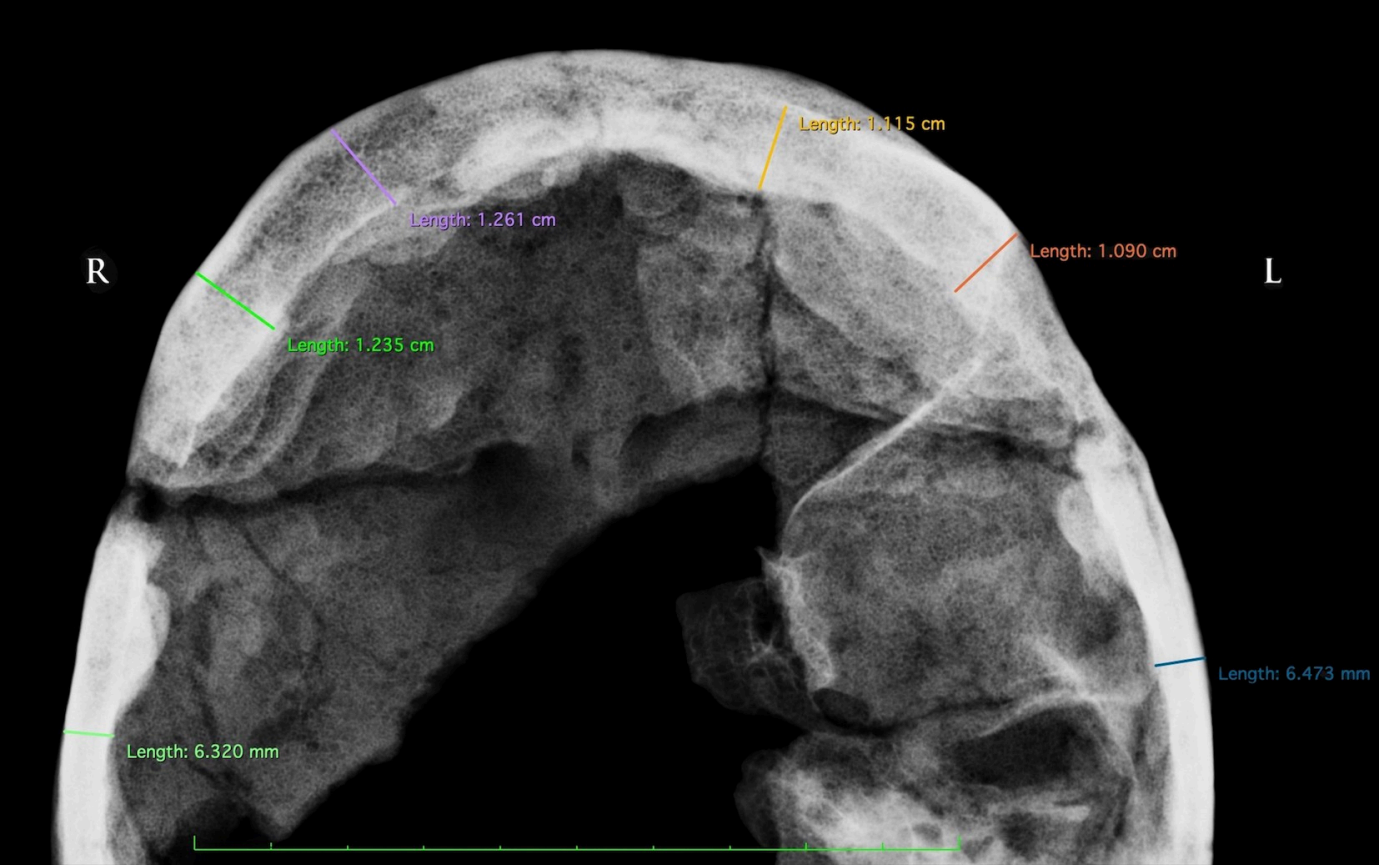
Postero-anterior X-ray with measurement of the thickness of the frontal and parietal bones indicating the hyperostosis. The obliteration of the frontal sinuses can be observed.

**Fig 7:**
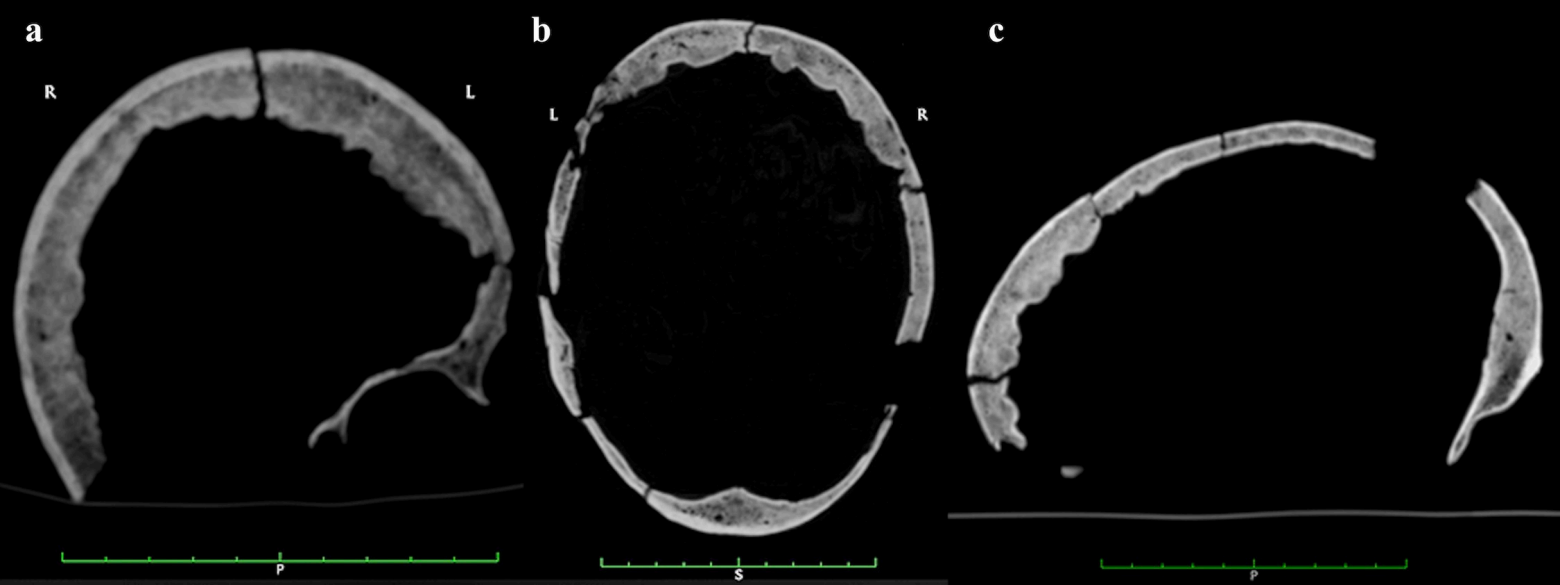
CT-scans in all projections: a. coronal view, b. axial view, c. sagittal view. The thickening and extent of the bony growth in the form of nodules protruding into the cranial cavity can be appreciated. A general thickening of the parietal and occipital bones can also be seen.

As early as during the restoration process a marked thickening of the cranial vault could be appreciated, as it would be confirmed by the radiological examination. This showed that the frontal sinuses are almost entirely obliterated by the bony growth, which filled their natural pneumatic spaces: this condition can be noticed more clearly in an antero-posterior projection radiograph (**Fig 6**) as well as on coronal, axial and sagittal CT-scans (**Fig 7A–7C**). Moreover, the entire skull is affected by thickening of the cranial tables, including at the occipital level (**Fig 7C**). The thickness of the frontal bone (inner table + diploe + outer table), measured at four different sites, yields an average of 11.75 mm. The thickness of the right parietal bone is 6.32 mm, and that of the contralateral is 6.47 mm.

Specifically in the frontal bone, the ectocranial plate retained its normal appearance, while the diploic space and endocranial plate were thickened.

**Fig 8:**
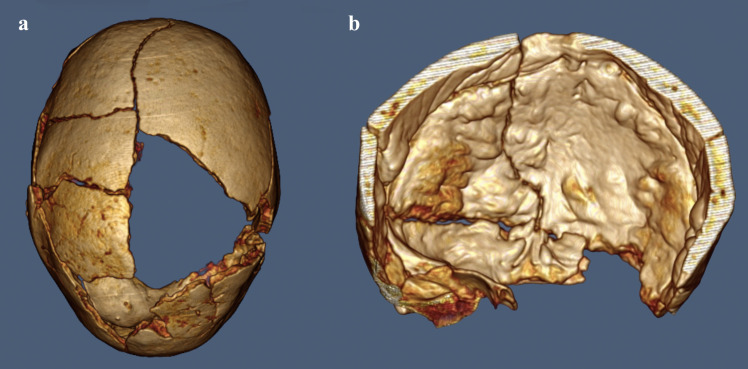
3D virtual reconstruction: a. superior view of the restored skull; b. coronal section of the frontal bone with the software *OsiriX Lite*.

The nodular appearance of the endocranial surface can also be properly evaluated on 3D virtual reconstructions of the skull (**Fig 8A and 8B**). The calculated endocranial volume results in 70.54 cm³ in the specimen with the endocranial alteration and 98.55 cm³ in the non-pathological skull adopted as a comparison.

**Fig 9:**
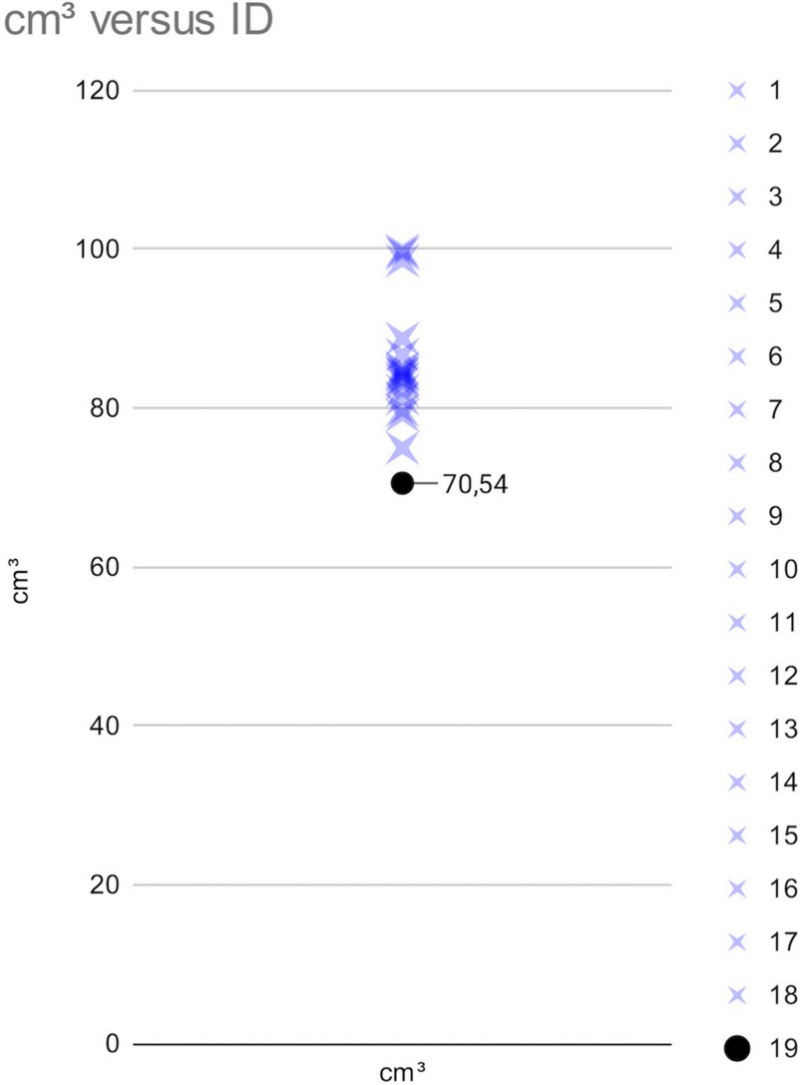
Graph showing the distribution of endocranial volumes (expressed in cm^3^) in the skull with HFI vs in the skulls used for comparisons.

Furthermore, even when more skulls are considered in comparative terms for statistical reasons, the reduction in endocranial volume is confirmed. Indeed, the volumetry of the donor’s skull used above was closer to 100 cm³ than the higher concentrations of volumes from the 18 skulls (average volume = 85.56 cm³ with SD ± 6.92). Additionally, it can be underlined that the skull with HFI was larger than the average of the donors, so that, even in the case of a larger skull, the endocranial volumetry was smaller than that of all the donors (**Fig 9**).

## Discussion and conclusion

*Hyperostosis frontalis interna* (henceforth HFI), whose aetiology is currently unknown–hormonal influence on bone growth being postulated–is often an incidental finding, with a prevalence in the general population between 5% and 12% and a predilection for the female sex [11]. HFI is defined as a remodelling of the inner plate of the frontal bone, typically presenting at the level of the frontal eminence of the endocranial plate, into a more cancellous morpho-phenotype, with midline, ex-suture, sparing, was described as being at the border between normal and pathological anatomy for the first time by Santorini and Morgagni in the 18^th^ century [12]. As Hawkins and Martin put it in 1965, ‘[d]espite a voluminous literature very little is known about hyperostosis frontalis interna. Thus its mode of formation and growth is unknown, the incidence among the general population is uncertain, and the question as to whether it is merely an anatomical anomaly or a pathological lesion remains un-answered.’ Much of that scientific uncertainty still holds true to this day [13].

From the anatomical point of view, at gross examination HFI is characterised by an increased volume and porosity of the inner plate and diploe of the frontal bone with nodular or sessile benign overgrowths in the inner aspect of the osseous surfaces; it may affect the bone in a focal or diffuse fashion, and normally it is present bilaterally and symmetrically. This condition can also extend to the parietal bones: if their involvement is complete, this presentation is named *hyperostosis frontoparietalis* [14]. However, as demonstrated by Hershkovitz and colleagues through electron microscopy, in HFI it is only the endocranial layer that changes in its morphology [15]. It should, nonetheless, be underlined that in the case presented here a generalised diploic thickening can be seen, which could point to a combination of HFI and cranial thickness as described by May and colleagues [16].

HFI has been rarely reported in bioarchaeological studies, most of the existing communications on this condition are in the form of single reports [15, 17].

The absence of involvement of the outer plate of the frontal bone is essential to make a differential diagnosis with some common metabolic bone diseases, such as rickets, acromegaly and Paget’s disease [15]. In addition, regarding neoplasms, meningioma, endosteal osteoma or osteosarcoma were excluded because they are rarely multifocal [12, 15]. The typically thickened inner table of the frontal bone is radiographically described as dense and spherical structures, as a sort of ‘bullets’, tending to protrude into the diploe and can also protrude into the cranial cavity like ‘string of pearls’ [12]. HFI can be associated with rare syndromes such as Morgagni’s syndrome (HFI, obesity, virilism), Stewart-Morel syndrome (HFI, obesity, mental disturbances), and Troell-Junet syndrome (HFI, acromegaly, toxic goitre, and diabetes mellitus) [12, 18–20].

Although these case reports often state that several clinical conditions seem to be associated with HFI, such as testosterone/estrogen dysmetabolism, psychiatric disease, obesity, diabetes, although in hazard/risk studies no significant differences were found between HFI and control groups, so that it should be considered an independent entity. Currently, there is not a univocal link between signs and symptoms and HFI, and the only evidence is about its reported association with elderly post-menopausal women [15, 21].

Very important in the study of this condition are also the anthropological examinations by Hershkovitz and colleagues, who investigated over 2,000 skulls from different geographical sites and historical periods, down to the 19^th^ century AD and belonging to various ethnic groups, which did not have signs of HFI, while HFI was identified in 24% (female) and 5% (male) of 1,700 skulls dated to the 20^th^ century [15]. This discrepancy was explained by the fact that high HFI prevalence is due to greater longevity, above all in women as a result of a long estrogen stimulation. In addition, the more pronounced longevity, as a result of improved life conditions could have been influenced by the environment [15]. To explain this phenomenon, some researchers have hypothesised that food availability during human evolution determines a metabolic rate increase, that leads to leptin level rise; leptin, for its part, controlling hypothalamic metabolic pathways, influence BMI, energy expenditure and adrenal tone [22].

The data derived from this multidisciplinary analysis allow us to confirm that the skull may indeed have belonged to a female senile individual, hence confirming the scant information found together with the skull fragments. From the pathological perspective and based on the literature summarised above, a confident retrospective diagnosis of HFI can be formulated, particularly in the light of the similarity with other cases from the scientific literarure with the endocranial layer described as showing a presentation compatible with the radiological findings already described by Hershkovitz and colleagues: ‘a hyperdense layer, a ballooned, vascularised area, and a thin cortical shell encapsulating it’ [15] (**Fig 4A–4C**). The condition appears advanced and severe considering that the thickness of the frontal bone is much greater than the width usually recorded in non-pathological skulls [23].

As far as a more precise classification of HFI is concerned, Hershkovitz and colleagues proposed four types of HFI, based on the quantity of bone involvement and extension, appearance, shape and location of the lesions [15]. Nikolic analysed the aspects and occurrence of different types of HFI, including the study 248 of deceased females, with HFI found in 45 of them (18.4%), and demonstrated that HFI has no correlation with age [24].

Based on these considerations, the case we presented can be reasonably catalogued as a severe Type D HFI (i.e. continuous nodular bony formations involving more than 50% of the endocranium of the frontal bone) of the classification by Hershkovitz and colleagues [15].

Compared to other morphological studies on HIF, particularly those on skeletal remains from anthropological and forensic contexts, in the present study a neuroanatomical approach, mediated by the above-seen generation of a brain endocast, allows for additional considerations on the presentation of this condition and its neuro-psychiatric correlations. The brain endocast shows a conspicuous atrophy of the woman’s brain at the level of the frontal pole of the cerebrum, with a particular involvement of the frontal lobe. Particularly affected by the inward growth of the endocranium is the prefrontal cortex which appears atrophic as a result of a mass-effect exerted by the bone tissue pushing onto the subjacent cerebral structure. Brodmann areas 9 and 10 appear to be the ones mostly affected by the process, with lesser, yet detectable involvement of areas 8 and 46. In the occipital lobe the HFI appears to be causing a limited reduction in volume of areas 17 and 18. Especially, Brodmann area 9 is involved in several functions including short-term memory, inductive reasoning, attributing intention, auditory verbal attention, empathy, etc [25]. It was reported to be affected in bipolar disorder [26]. In 1953, Notkin considered several types of interplay between cranial changes and reported neuropsychiatric symptoms, one of which was the possibility that the psychosis he had diagnosed in some patients ‘could be the result of interference with the function of the brain by direct action of the structural bone changes’, particularly as he had observed in one case showing ‘definite neurologic signs’, hence suggesting a mechanism of direct action onto the brain, although he also considered more possibilities such as the fact that both psychotic and bone changes could result from common unknown aetiologic factors [27]. Notkin noted that in favour of this last possibility was the evidence that some of the cases analysed in his study ‘started with various functional types of psychosis’ and that they ‘finally changed into organic reactions’, whereas other cases ‘showed signs of organic deterioration practically from the beginning’ [27]. A similar causative correlation was more recently proposed by Gilbert et al. in 2012 [28], who, just like in the skeletal case presented here, studied a type-D presentation of HFI, which had even reached a much more advanced stage affecting not only the prefrontal cortex but also the frontal one. The major advantage of Gilbert’s study was that the scientific team could perform an MRI in an *intra vitam* patient and could access this postmenopausal woman’s clinical file, which included a diagnosis of psychiatric disorders inclusive of a ‘tendency towards paranoia, emotional indifference and aggressiveness’. Interestingly Gilbert and colleagues highlighted ‘a progressive worsening in behaviour’, which can be postulated to be compatible with the contextual growth of the inner cranial table [28]. A similar anatomo-clinical correlation can be reasonably postulated since in the case discussed here an initial alteration of the endocranial surface was noted in the areas of the frontal bone close to the coronal suture and in the more frontal part of the parietal bones, hence being the harbinger of further inward expansion and involvement of the frontal cortex.

Unfortunately, from the purely clinical perspective, the detrimental loss of information on the woman whose skull has been the object of the present investigation does not allow us to make any confident attempt at precisely correlating anatomical alterations with behavioural changes and neurological symptoms, yet this approach shows how in forensic and anthropological cases the study of the brain endocast can help scientists retrospectively understand the impact of cerebral changes induced by describable endocranial alterations. In addition, reinforcing some previous clinical case reports, this our analysis suggests that a larger neuroradiological study ought to be implemented in living patients with known neuropsychiatric diagnoses in order to monitor and stage the progression of this complex endocrine-osteological-neurological entity. This could be matched by more *post-mortem* assessments in both forensic and anatomical contexts. Once more, the study of the dead could help shed light on the nature of pathological processes, and ultimately help the living.

### Ethical statement

This study initially started after being authorised by the then director of the Section of Legal Medicine of the University of Foggia in 2018, co-author of this study Prof. Pietrantonio Ricci. This case falls under the umbrella of the Italian Police Mortuary Rules (DPR 09.10.1990 n° 285, art. 83). For the skull with HFI no consent was applicable. The present study also used a bank of anonymised CT scans of individuals of different ancestry (virtual donors), being Brazilians, Moldovans and Malaysians, under the protocol USMKK/PPP/JEPeM [259.3(2)], which received ethical approval from the Human Research Ethics Committee, Universiti Sains Malaysia. Co-author of this article Cicero Moraes confirms that he was authorized to used data from their dataset for his own research, including the present study.

## Supporting information

S1 Video (MOV)

## References

[1] Ferembach D, Schwidetzky I, Stloukal M. Recommandations pour déterminer l’âge et le sexe sur le squelette. Bull Mem Soc Anthropol Paris. 1979;6(1): 7–45.

[2] Meindl RS, Lovejoy CO. Ectocranial suture closure: a revised method for the determination of age at death based on the lateral-anterior sutures. Am J Phys Anthropol. 1985;68: 57–66. 4061602 10.1002/ajpa.1330680106

[3] Moraes C, Dornelles R, da Rosa E. OrtogOnBlender—O que é e Aspectos Técnicos. Figshare, 2020. 10.6084/m9.figshare.12923729.v1

[4] Varotto E, Magro MT, Brancato R, Lubritto C, Memeo L, Galassi FM. Unique Osteoid Osteoma of the Frontal Sinus From the Late Roman Empire. J Craniofac Surg. 2019;30(4): 965–966. doi: 10.1097/SCS.0000000000005312 30817534

[5] Galassi FM, Varotto E, Angelici D, Picchi D. Further Paleoradiological Evidence of Frontal Sinus Osteoma in Ancient Egypt. J Craniofac Surg. 2020;31(3): 604–605. doi: 10.1097/SCS.0000000000006240 32195832

[6] Habicht ME, Bianucci R, Buckley SA, Fletcher J, Bouwman AS, Öhrström LM, et al. Queen Nefertari, the Royal Spouse of Pharaoh Ramses II: A Multidisciplinary Investigation of the Mummified Remains Found in Her Tomb (QV66). PloS One 2016;11(11): e0166571. doi: 10.1371/journal.pone.0166571 27902731 PMC5130223

[7] Seiler R, Habicht ME, Rühli FJ, Galassi FM. First-time complete visualization of a preserved meningeal artery in the mummy of Nakht-ta-Netjeret (ca. 950 BC). Neurol Sci. 2019;40(2): 409–411. doi: 10.1007/s10072-018-3565-1 30215156

[8] Moraes C, Dornelles R, da Rosa E. Sistema de Reconstrução de Tomografia Computadorizada Baseado no Slicer 3D e no DicomToMesh. Figshare, 2021. 10.6084/m9.figshare.13513890.v1.

[9] https://docs.blender.org/manual/en/latest/modeling/modifiers/generate/booleans.html (last accessed on 27th February 2021).

[10] Abdullah JY, Saidin M, Rajion ZA, Hadi H, Mohamad N, Moraes C, Abdullah JM. Using 21st-Century Technologies to Determine the Cognitive Capabilities of a 11,000-Year-Old Perak Man Who Had Brachymesophalangia Type A2. Malays J Med Sci. 2021;28(1): 1–8. doi: 10.21315/mjms2021.28.1.1 33679214 PMC7909344

[11] Western AG, Bekvalac JJ. Hyperostosis frontalis interna in female historic skeletal populations: Age, sex hormones and the impact of industrialization. Am J Phys Anthropol. 2017;162(3): 501–515. doi: 10.1002/ajpa.23133 27901271

[12] She R, Szakacs J. Hyperostosis Frontalis Interna: Case Report and Review of Literature. Ann Clin Lab Sci. 2004;34(2): 206–208. 15228235

[13] Hawkins TD, Martin L. Incidence of Hyperostosis Frontalis Interna in Patients at a General Hospital and at a Mental Hospital. J Neurol Neurosurg Psychiatry. 1965;28(2): 171–174. doi: 10.1136/jnnp.28.2.171 14285657 PMC495878

[14] Tripathi M, Bal C, Damle NA, Singhal A. Hyperostosis fronto-parietalis mimicking metastasis to the skull: Unveiled on SPECT/CT. Indian J Nucl Med. 2012;27(4): 272–273. doi: 10.4103/0972-3919.115406 24019665 PMC3759096

[15] Hershkovitz I, Greenwald C, Rothschild BM, Latimer B, Dutour O, Jellema JM, et al. Hyperostosis frontalis interna: an anthropological perspective. Am J Phys Anthropol. 1999; 109: 303–325. doi: 10.1002/(SICI)1096-8644(199907)109:3<303::AID-AJPA3>3.0.CO;2-I 10407462

[16] May H, Mali Y, Dar G, Abbas J, Hershkovitz I, Peled N. Intracranial volume, cranial thickness, and hyperostosis frontalis interna in the elderly. Am J Hum Biol. 2012;24(6): 812–819. doi: 10.1002/ajhb.22325 23012133

[17] Ruhli FJ, Henneberg M. Are hyperostosis frontalis interna and leptin linked? A hypothetical approach about hormonal influence on human microevolution. Med Hypotheses. 2002;58: 378–381. doi: 10.1054/mehy.2001.1481 12056872

[18] Chaljub G, Johnson RF 3rd, Johnson RF Jr, Sitton CW. Unusually exuberant hyperostosis frontalis interna: MRI. Neuroradiology 1999;41: 44–45. doi: 10.1007/s002340050703 9987768

[19] Schneeberg NG, Woolhandler G, Levine R. The clinical significance of hyperostosis frontalis interna. J Clin Endocrinol. 1947;7(9): 624–635. doi: 10.1210/jcem-7-9-624 20264653

[20] Dann S. Metabolic craniopathy: a review of the literature with report of a case with diabetes mellitus. Ann Intern Med. 1951;34: 163–202. 14790546 10.7326/0003-4819-34-1-163

[21] Fakoya A, Heymans J, McCrary A, Rodriguez O, Cardona A, Afolabi A, et al. Hyperostosis Frontalis Interna: A Case Report. J Health Sci. 2020;10(2): 170–172.

[22] Ruhli FJ, Boni T, Henneberg M. Hyperostosis frontalis interna: archaeological evidence of possible microevolution of human sex steroid? HOMO 2004;55: 91–99. 15553271 10.1016/j.jchb.2004.04.003

[23] Ruan J, Prasad P. The effects of skull thickness variations on human head dynamic impact responses. Stapp Car Crash J. 2001;45: 395–414. doi: 10.4271/2001-22-0018 17458755

[24] Nikolic S, Djonić D, Zivković V, Babić D, Juković F, Djurić M. Rate of Occurrence, Gross Appearance, and Age Relation of Hyperostosis Frontalis Interna in Females. A Prospective Autopsy Study. Am J Forensic Med Pathol. 2010;31(3): 205–207. 20177366 10.1097/PAF.0b013e3181d3dba4

[25] Goel V, Gold B, Kapur S, Houle S. The seats of reason? An imaging study of deductive and inductive reasoning. Neuroreport. 1997;8(5): 1305–1310. doi: 10.1097/00001756-199703240-00049 9175134

[26] Brooks JO 3rd, Bearden CE, Hoblyn JC, Woodard SA, Ketter TA. Prefrontal and paralimbic metabolic dysregulation related to sustained attention in euthymic older adults with bipolar disorder. Bipolar Disord. 2010; 12(8): 866–874. doi: 10.1111/j.1399-5618.2010.00881.x 21176034

[27] Notkin J. Frontal bone hyperostosis in psychoses; a clinical study. Am J Psychiatry 1953; 109(12): 929–935. doi: 10.1176/ajp.109.12.929 13050820

[28] Gilbert T, Ait S, Delphin F, Raharisondraibe E, Bonnefoy M. Frontal cortex dysfunction due to extensive hyperostosis frontalis interna. BMJ Case Rep. 2012;2012: bcr0720114439. doi: 10.1136/bcr.07.2011.4439 22665704 PMC4543109

